# Reproduction reimagined

**DOI:** 10.1016/j.xfre.2021.11.004

**Published:** 2021-11-09

**Authors:** Richard J. Paulson

**Affiliations:** Division of Reproductive Endocrinology & Infertility, Department of Obstetrics & Gynecology, Keck School of Medicine, University of Southern California, Los Angeles, California

Those who attended the 2021 Scientific Congress and Expo of the American Society for Reproductive Medicine were treated to a dazzling array of lectures and presentations about the future of our field. The theme “Reproduction Reimagined” was aptly chosen, and it was gratifying to note the allusion to an article with the same name that graced our inaugural issue (1). In that special contribution, Adashi and Cohen (1) presented and discussed the very issues that were presented in this year’s meeting: in vitro gametogenesis, assisted same-sex reproduction, and germline remediation of genetic diseases. Science is truly marching on. Those of us who have been in this field for some time have grown accustomed to the regular appearance of dramatic and game-changing advances. It is remarkable and reassuring that the pace of their appearances does not seem to be slowing down.

In vitro gametogenesis is particularly appealing. How much fun will it be to tell future generations of infertility specialists that, yes, we had to stimulate ovaries to get eggs. What if the patient did not respond very well? Then we did not get many eggs. Did eggs from women in their 40s not produce embryos with limited implantation potential? That is correct; such pregnancies were not very common. It is very tempting to imagine a large number of eggs derived from induced pluripotent stem cells, which would yield a sufficiently large number of euploid blastocysts, ensuring that patients would be able to complete their family. The choice of if, when, and with whom to have a family would finally be available to everyone.

How far from completing this fantasy are we? With DNA undergoing demethylation and subsequent remethylation during gametogenesis and early embryo development, the molecular details of an early conceptus are extremely complex. How will we ascertain that in vitro-derived gametes will follow the same (safe) developmental path? Same-sex reproduction brings up the issue of imprinted genes, which express only the maternal or paternal allele. Which molecular tricks will be necessary to achieve correct gene imprinting and avoid Angelman or Beckwith-Wiedemann syndrome? For the germline remediation of genetic diseases, CRISPR-Cas9 presents an amazing opportunity to edit genetic mutations and, thus, eliminate specific inherited diseases. However, its ability to modify only the one specific target gene is limited, and off-target effects may produce unknown and potentially dangerous errors.

As Yogi Berra said, “It’s tough to make predictions, especially about the future” (2). Even if these technologies are not around the corner, it is important that we continue the quest for their discovery. We may find answers and will quite likely learn a lot of other, potentially useful (perhaps unanticipated), things along the way. As long as the research does not involve human embryos, federal funding is (at least theoretically) available to support this research. Somewhere along the way, human embryos will have to be investigated, and researchers may have to rely on the American Society for Reproductive Medicine Research Institute to help underwrite investigations that the government will not.

Perhaps, while we wait for new technologies to become a reality, we should consider reimagining the use of the one technology that is already a reality today: oocyte cryopreservation. Oocyte cryopreservation has been around long enough for us to know that it works (3, 4). While we wait for advances in in vitro gametogenesis, oocyte cryopreservation offers a temporary fix to the problem of declining fertility, at least for some patients. In current practice, oocyte cryopreservation is often seen as a last-ditch effort to preserve fertility for those who are about to lose ovarian function. For example, it has been suggested that the greatest improvement in the probability of live birth occurs when oocyte cryopreservation takes place at the age of 37 years (5). This may be true for a given population, but should we instead be considering the maximum benefit of oocyte cryopreservation to the individual? Younger women produce higher numbers of higher-quality eggs. For maximum benefit to the individual, oocyte cryopreservation should likely be performed a decade earlier in life, when a single cryopreservation cycle can produce enough eggs for 2 (or more) live births. As we reimagine the future, let us not ignore the technology we already have at our disposal. Let us also reimagine the present.
